# Improving biomarker list stability by integration of biological knowledge in the learning process

**DOI:** 10.1186/1471-2105-13-S4-S22

**Published:** 2012-03-28

**Authors:** Tiziana Sanavia, Fabio Aiolli, Giovanni Da San Martino, Andrea Bisognin, Barbara Di Camillo

**Affiliations:** 1 Department of Information Engineering, University of Padova, via G. Gradenigo 6/B, 35131 Padova, Italy; 2 Department of Pure and Applied Mathematics, University of Padova, Via Trieste 63, 35121, Padova, Italy; 3 Department of Biology, University of Padova, Via G. Colombo 3, 35121, Padova, Italy

## Abstract

**Background:**

The identification of robust lists of molecular biomarkers related to a disease is a fundamental step for early diagnosis and treatment. However, methodologies for biomarker discovery using microarray data often provide results with limited overlap. It has been suggested that one reason for these inconsistencies may be that in complex diseases, such as cancer, multiple genes belonging to one or more physiological pathways are associated with the outcomes. Thus, a possible approach to improve list stability is to integrate biological information from genomic databases in the learning process; however, a comprehensive assessment based on different types of biological information is still lacking in the literature. In this work we have compared the effect of using different biological information in the learning process like functional annotations, protein-protein interactions and expression correlation among genes.

**Results:**

Biological knowledge has been codified by means of gene similarity matrices and expression data linearly transformed in such a way that the more similar two features are, the more closely they are mapped. Two semantic similarity matrices, based on Biological Process and Molecular Function Gene Ontology annotation, and geodesic distance applied on protein-protein interaction networks, are the best performers in improving list stability maintaining almost equal prediction accuracy.

**Conclusions:**

The performed analysis supports the idea that when some features are strongly correlated to each other, for example because are close in the protein-protein interaction network, then they might have similar importance and are equally relevant for the task at hand. Obtained results can be a starting point for additional experiments on combining similarity matrices in order to obtain even more stable lists of biomarkers. The implementation of the classification algorithm is available at the link: http://www.math.unipd.it/~dasan/biomarkers.html.

## Background

Analysis of gene expression from microarray experiments has been widely used for the development of new physiological hypotheses useful for answering to both diagnostic and prognostic questions. In the last decade, supervised classification analysis has experienced a large diffusion to address this task and several different methods like discriminant analysis, random forests and support vector machines among others, have been used on gene expression data, especially in cancer studies [1,2].

In these studies, the biological interest is mainly focused on biomarker discovery, i.e. in finding those genes and proteins which can be used as diagnostic/prognostic markers for the disease. Biomarkers provide useful insight for a deeper and more detailed understanding of the biological processes involved in the specific pathology and might represent the targets for drug development [3]. Although high accuracy is often achieved in classification approaches, biomarker lists obtained in different studies for the same clinical type of patients have few genes in common [4,5], whereas biomarker reproducibility is fundamental for clinical and pharmaceutical applications. Several works have recently pointed out that high reproducibility of biomarker lists is equally important as high classification accuracy [6,7].

In general, there are two stability issues arising in gene expression classification and analysis. Firstly, since training data are often scarce, predictive models obtained from different datasets can be extremely different. Secondly, since the number of features is generally very high, then features can be combined in many different ways to give solutions able to explain the data. As a consequence of this, many possible sets of features can be considered relevant to the task and equally good in terms of the accuracy. This characteristic makes the process of selecting the set of relevant features for a classification task a very hard problem.

Bootstrap methods have been demonstrated helpful in addressing the first issue. In these approaches different classifiers are generated and different features (lists of biomarkers) are selected on different splits of data and the results are somehow averaged [8,9], thus preserving a high ranking only to those features that are consistently the most discriminating features over the splits [10,11]. However, this method does not solve the problem of the instability due to the high number of features. In fact, the crucial problem is that the classification task is under constrained. To address this issue, additional information available on the relationships between genes should be used to improve the stability with respect to the features of the classifiers. The basic idea of this strategy is to take into account the complex gene relationships, instead of considering genes as independent features. Several efforts in this direction have been recently presented in the literature. In [12], pathway information has been incorporated into the biomarker discovery process using available protein-protein interaction networks and considering subnetworks as features. Logistic regression models have been applied on expression profiles of two cohorts of breast cancer patients and results have been assessed in terms of both agreement between subnetworks identified in the two datasets and classification accuracy. In [13-17] topological properties of Kyoto Encyclopedia of Genes and Genomes (KEGG) pathways [18] or networks reconstructed from gene expression data have been used to constrain the learning process. In particular, [16,17] use regularization and integrate prior knowledge defining KEGG pathway based penalty terms. The use of Gene Ontology (GO) [19] as prior information has been explored in [20], where the authors propose a classification model based on functional groups of genes. All the above methods have focused on prediction performance, without considering in a systematic way the stability issue. Recent works have started considering the problem of biomarker list stability [21], but an overview of the ability of different sources of biological knowledge to improve the reproducibility of the results is not already available in the literature.

Our work addresses the integration of prior knowledge in the learning process and, differently from previous works, compares the performance of different sources of prior knowledge. In particular, we propose a standardized way to incorporate in the kernel different types of biological knowledge like functional annotations, protein-protein interactions, and expression correlation among genes, with the only constraint that the information is codified by a similarity matrix. The feature space is then transformed such that the more similar two features are, the more closely they are mapped. Similarity matrices are defined using metrics which are specific for each type of biological information used: semantic similarities [22] for the annotations on GO; topology-based similarity measures [23,24] for protein-protein interactions (PPI) extracted from Human Protein Reference Database (HPRD) [25]; pair-wise correlation and mutual information for gene expression data. A linear classifier resembling the Bayes Point Machine [26] is used as classification tool. The vector of weights produced by this algorithm is used to rank the features and obtain a list of biomarkers.

Differently from approaches that integrate different datasets [27] by combining kernels [28] to improve classification performance and robustness of the results thus considering a different and maybe complementary aspect of the problem, our approach addresses the integration of prior knowledge in the learning process. It provides a standardized way to incorporate different types of biological knowledge in the kernel, with the only constraint that the information is codified by a similarity matrix, thus it can be used with any kernel method.

As above mentioned, in this work we also compare the performance of different sources of prior knowledge and evaluate the performance using three real datasets from different studies exploring the same clinical classification task. The assessment of the results obtained for different similarity matrices is based on the trade-off between predictive accuracy and feature ranking stability, measured using the Canberra distance [7]. In fact, the introduction of constraints in the feature space might lead to robust biomarker lists but poor discrimination between the classes. Finally, we have evaluated the ability of different biological information to map the features on new feature spaces where the classes are more naturally separable.

## Methods

### Gene expression data

Publicly available data from three breast cancer microarray studies were collected from Gene Expression Omnibus (GEO) database [29] with accession numbers: GSE2990 [30], GSE3494 [31] and GSE7390 [32]. Datasets were all hybridized using Affymetrix U133 Genechips™ (HG-U133A). Breast cancer has been extensively studied in the literature and the Estrogen receptor (ER) status is the most important prognostic factor as indicator of response to endocrine therapy [33]. Estrogen receptor 1 (ESR1) is the gene more directly associated with ER status and can mask other potential descriptors of the underlying pathophysiology [34]; therefore, probesets related to ESR1 were removed from all datasets. Only tamoxifen-untreated subjects were selected. Since there are subgroups of samples belonging to multiple datasets, redundant subjects were removed. Quality assessment of the raw data from each dataset was performed using the arrayQualityMetrics package in Bioconductor [35]. Any array failing quality controls on MA plots, box-plots and between-array distances was not considered. Affymetrix chip definition files were used to annotate the arrays, resulting in 22207 features. Gene expression intensity signal was derived and normalized independently for each dataset using robust multiarray average (RMA) algorithm [36]. The resulting datasets are described in Table 1.

**Table 1:** Breast cancer datasets used for the classification

Datasets	Samples	ER+ samples	ER- samples
GSE2990	116	83	33
GSE3494	155	131	24
GSE7390	152	103	49

### Integration of prior knowledge in the learning process

Expression data are given as very high dimensional vectors of measurements. The high dimensionality makes the task of biomarkers discovery very hard. This is especially due to the fact that the task is under constrained. In this paper, we propose to perform a linear transformation of the examples (i.e. the biological samples) in a way that classifiers computed on transformed examples have a higher stability, hopefully preserving the accuracy. This transformation is made by using prior biological information about genes in a way to maintain the structure of the problem.

In this section we first introduce linear classifiers and some relevant facts about the embedding of data into feature spaces. Then, we describe our intuition and describe an algorithm that implements it.

In the following we denote by $\left\{{\vec{x}}_{1}\text{, }...\text{, }{\vec{x}}_{M}\right\}$ the examples, i.e. the *N *dimensional vectors of expression data obtained for *M *subjects, where *N *is the number of genes. Each example has associated a binary label *y_m _*(*m *= 1,..., *M*) having values in {-1,+1}.

#### Linear classifiers

We focus on linear classifiers which are simple and generally perform well on gene expression analysis. Given a linearly separable classification task, there are in general infinitely many linear classifiers (hyper-planes) that can correctly classify the examples. This set is commonly called the *version space*. When the number of features is very high, the version space tends to have a large volume. Formally, the version space for linear classifiers can be defined as:

(1) $$V=\{\vec{w}|y_{m}(\vec{w}\cdot {\vec{x}}_{m})>0,\;for\;each\;m=1,...M,\left\|\vec{w}\right\|=1\}$$

Without any loss in generality, we consider weights of unitary norm. A very popular algorithm to find a linear classifier which correctly separates the training examples (i.e. an element of the version space) is the Perceptron algorithm [37] which can be briefly described as in the following. We assume the training vectors *x *and *w *are of size *n*, and *w *is initially set to the zero vector. The algorithm runs in epochs. On each epoch all the training examples *x_i_*, for *i = 1,.., M*, are presented to the algorithm and the vector *w *is updated whenever the associated classifier makes a mistake on *x*_i_, i.e. if *(y_i _sign(w · x_i_) ≤ 0) *then *w = w+y_i_x_i_*. When the training set is linearly separable, the perceptron is guaranteed to eventually converge to a vector (hyper-plane) which correctly separates the training data, i.e. the solution is an element of the version space.

It can be shown that other kernel based algorithms, like for example the hard version of Support Vector Machines (SVM), whose description is beyond the scope of the paper, also have solutions in the version space. In the particular case of SVM this solution is in fact unique and is the one which maximizes the margin on the training set [38]. As shown in [26] the center of mass of the version space, the so called Bayes point (Bp), would be the optimal choice, even better than SVM (which by the way can be considered an approximation of the Bp), with nice theoretical properties in terms of its generalization ability. An algorithm that approximates this optimal Bp solution, the so called Bayes point machine, has been proposed, which considers the average of the solutions of several runs of the perceptron. Our algorithm, which is presented in the following, is based on a variant of the Bayes point machine.

Note that when a feature space is characterized by high dimensionality and the features are considered independent, i.e. there are many more variables (features) than constraints (examples), the task is under constrained. This often implies that the version space volume is large and can change extremely both in form and size depending on which examples are used for training. It is clear that this produces instability. We will see in the following how to add available domain knowledge to introduce structural constraints in the problem in order to improve robustness of a linear classifier.

#### Feature ranking

The values *w_i _*of a linear classifier represent the degree of importance and the bias that a given feature *i *provides to the decision. High positive (negative) values tell us that such feature is important to classify an instance as positive (negative). For this reason, the absolute value of the weights can also be used as a criterion for feature ranking.

#### Similarity matrix integration

When prior knowledge is available providing information about gene-gene similarity, this knowledge can be effectively used by mapping examples into a feature space where linear solutions preserve these similarities.

Consider a linear transformation of the data via a matrix *P*, i.e. *ϕ*(x) = Px. Now, can we say something about the desirable properties of *ϕ *which make the task of discriminating positive versus negative examples simple enough in the target space? It is well known that a measure of the goodness of an embedding is the ratio between the maximal norm *R*, the highest norm (or length) of any example *x_m_*, and the margin *γ *of the examples, namely *G = (R/γ)^2^*. For separable data, the margin is defined as the distance between the optimal separating hyper-plane (SVM) and the examples. In the case of perceptron classifiers, the value *G *is also related to the number of mistakes the perceptron algorithm makes to converge [37]. These considerations seem to indicate that the margin of transformed examples should be large in order to get high performance. However, when the expected margin (or equivalently, the expected volume of the version space) is too large, it generally leads to unstable solutions for small datasets. A solution, which represents a trade-off between these two (apparently) opposite goals, is to choose an embedding of data where norm of vectors are as small as possible but data remain linearly separable. Specifically, we propose to make a linear embedding of data via a bi-stochastic matrix. We focus on stochastic matrices because they have the property to map vectors × into shorter ones (compression) and thus to make the maximal norm *R *of target examples smaller (this is due to the fact that the eigenvalues of a stochastic matrix are all in [0, 1]). As we have previously seen, this together with large margin solutions guarantees a good performance of the embedding.

Let *S *be a symmetric similarity matrix with elements in [0, 1] with 1's in the diagonal, the associated stochastic matrix *P *is obtained as in the following:

(2) $$P=D^{-1}\left(I+\alpha \left(S-I\right)\right)$$

where *I *is the identity matrix, *D *is a diagonal matrix with elements corresponding to sums of elements in the rows/columns of *(I+α(S-I))*, and α > 0 is a tuning parameter. Note that when α = 0, we have *P = I *and the feature space coincides with the original space. The parameter α is fixed according to the best stability performance, measured by the Canberra distance (Equation 15).

Now, let be given a perceptron-like solution in the target space, then the weight vector can be expressed as a weighted sum of the examples in feature space, namely $\vec{w}=\sum \beta_{m}\varphi \left({\vec{x}}_{m}\right)$, and the following holds:

(3) $$\begin{array}{c} \left|w_{i}-w_{j}\right|=\left|\sum_{m}\beta_{m}\sum_{k}P_{ik}x_{mk}-\sum_{m}\beta_{m}\sum_{k}P_{jk}x_{mk}\right|=\\ =\left|\sum_{m}\beta_{m}\sum_{k}\left(P_{ik}-P_{jk}\right)x_{mk}\right|=\\ =\left|\sum_{k}\left(P_{ik}-P_{jk}\right)\sum_{m}\beta_{m}x_{mk}\right|=\\ =\left|\left({\vec{P}}_{i}-{\vec{P}}_{j}\right)\cdot h\right|\leq c∥\left({\vec{P}}_{i}-{\vec{P}}_{j}\right)∥ \end{array}$$

where *c ≥ 0 *is a constant which does not depend on indices *i *and *j*. Thus, we can see the matrix *P *as a coding matrix for genes. Specifically, the *i-th *gene is codified by *P_i_*. This result shows that when two genes have similar codes, the difference in the weight vector cannot be too large.

It is important to note that this result does not imply that the same gene will have the same position in the ranking generated by two independent experiments, i.e. that the same biomarkers will be selected. The result above simply means that the relative position of two similar genes will be similar in the two experiments. However, if the matrix *P *contains reliable information, this should hopefully produce similar lists of biomarkers.

#### Classification algorithm and biomarker list generation

The proposed algorithm is based on the perceptron algorithm and resembles the Bayes point machine. The algorithm starts by mapping data using the matrix *P*. The transformed data are standardized by subtracting from each gene expression value its mean across the samples and dividing by its standard deviation. Then, data are randomly split (70% training, 30% test) for a number *T *= 1000 of times. For each one of these splits a run of the perceptron algorithm is performed on its training data (to increase randomization data are also shuffled before each perceptron epoch). Thus, for each split *t*, a weight vector *w_t _* is obtained and normalized to unitary norm. For each split, the accuracy *a_t _*is also evaluated with respect to the test partition. The final solution is obtained as the average of weight vectors *w_t_*, i.e. *W *= AVE(*w_t_*).

Note that the expected accuracy of *W *on new unseen examples can also be estimated by using available data with the following method. Let *Q *be the design matrix with entries *Q_tm _*= 1 if the example *x_m _*is in the training partition of split *t*, and 0 otherwise. For each example *x_m _*a predictor *W*(*m*) = AVE(*w_t_*) is built using just the weights *w_t _*such that *Q_tm _*= 0, i.e. we take the average of the weight vectors for the construction of which the example *x_m _*was not used. Finally, the classifier *W*(*m*) is tested against *x_m_*. It is easy to see that the accuracy we observe applying this method on all available data is an estimate of the expected accuracy of *W*. The list of biomarkers returned by the algorithm is the list of genes ordered according to the absolute value of their correspondent value in *W*.

The method described above can also be seen as a leave-one-out estimate of the accuracy. However, the same method can be easily adapted to a (*k*-fold) cross-validation type of analysis. In this case, the overall procedure would change as in the following: (i) Split data in *k *sets *X_1_,.., X_k_*; (ii) Train models *W_1_,.., W_m_*, where *W_t_*, *t *= 1,..,*k*, is learned on the set *X\X_t_*, with the method presented above, and get the accuracy *ACC*(*X_t_*) on the set *X_t_*; (iii) Evaluate the overall accuracy as the average of these partial accuracy estimates.

The advantage of using a *k*-fold type of analysis instead of the leave-one-out type of analysis is its lower variance for small samples. The disadvantage is that the method is more computational demanding. We have done some experiments using both methods and we have not observed big differences in the obtained results with our data.

### Similarity matrices

Three different kinds of data were considered as prior knowledge to be integrated in the feature ranking: 1) Gene Ontology functional annotations; 2) the network of protein-protein interactions; 3) gene expression profiles from a collection of breast cancer studies. All these data were used to calculate different kinds of similarity measures *s_ij _*between pairs of features *i *and *j *based on:

• Semantic similarity of functional annotations;

• Topological similarity in the network of protein-protein interactions;

• Correlation between gene expression profiles.

The corresponding similarity matrix *S *for *N *variables is the symmetric *N*·*N *matrix whose element *s_ij _*refers to the similarity between the features *i *and *j*.

In the following, the methods for codifying the three types of prior knowledge into the corresponding similarity matrices are described in details. Since in this work we are considering Affymetrix data, indexes *i *and *j *refer to probesets. What follows can be easily generalized to consider genes or proteins. Each subsection first describes the biological information and then illustrates the metrics used to generate the corresponding similarity matrix.

#### Semantic similarity

Gene Ontology (GO) is the most widely used annotation database that collects biological information on gene products. This controlled vocabulary consists of three independent categories: molecular function, biological process and cellular component [19]. GO terms are organized in a directed acyclic graph (DAG) in which each node corresponds to a GO term. Each node may have multiple parents: nodes farther from the root (high level nodes) correspond to more specialized terms, nodes closer to the root (low level nodes) to less specialized terms, thus implying that genes annotated with a specific node are also annotated with every ancestor of that node (true path rule).

In this work molecular function and biological process GO annotations related to the probesets were downloaded from NetAffx database [39], while the DAG structure was extracted from the Bioconductor package GO.db.

Semantic similarity was used to assess the degree of relatedness between two features by assigning a metric based on the likeness of the semantic content of their GO annotation. An information-theoretic method, based on the concept of Information Content (*IC*), was adopted [40].

The *IC *for the GO term t is defined as:

(4) $$IC\left(t\right)=-\text{log}\left(\frac{freq\left(t\right)}{freq\left(root\right)}\right)$$

i.e. the negative logarithm of the ratio between the frequency of the term *t*F in a corpus of annotations (i.e. the number of times the term t and each of its descendants occur in GO annotation) and the frequency of the root term (corresponding to the sum of the frequencies of all GO terms). The *IC *decreases monotonically when moving from the leaves toward the root node (*IC *= 0). The intuition behind the use of the IC is that the more probable a concept is, the less information it conveys.

The Best-Match Average (BMA) approach [41] was used to calculate the semantic similarity scores *s_ij _*between two features *i *and *j*:

(5) $$s_{ij}=\frac{\frac{1}{\left|GO_{i}\right|}\underset{t\in GO_{i}}{\sum }{\text{max}}_{u\in GO_{j}}Sim_{Lin}\left(t,u\right)+\frac{1}{\left|GO_{j}\right|}\underset{u\in GO_{j}}{\sum }{\text{max}}_{t\in GO_{i}}Sim_{Lin}\left(u,t\right)}{2}$$

where *GO_i _*and *GO_j _*are the groups of GO terms *t *and *u *associated to the features *i *and *j*, respectively and *Sim_Lin_(t, u) *is the Lin's similarity measure [42], which exploits the *IC *of the two GO terms *t *and *u *to generate normalized similarity measures in the range [0, 1], according to the following equation:

(6) $$Sim_{Lin}\left(t,u\right)=\frac{2IC\left(MICA\left(t,u\right)\right)}{IC\left(t\right)+IC\left(u\right)}$$

where *MICA *indicates the most informative common ancestor. The BMA approach (Equation 5) is able to robustly assess the global similarity between two features also when they are annotated to a different number of GO terms, since it considers both the GO terms they share and the GO terms in which the features differ, but only the most similar ones are matched [22].

#### Topological similarity

Topological information on PPI was extracted from HPRD [25]. This repository contains manually curated scientific information pertaining to the biology of most human proteins and is the database that includes most human protein-protein interactions, as shown in [43]. The 22207 features in our datasets were mapped into 9521 proteins using RefSeq identifiers; this resulted in 37080 interactions. Since different proteins can be associated to different probesets, the value of the similarity score *s_ij _*between probesets *i *or *j *was obtained by averaging the similarity scores of the associated proteins:

(7) $$s_{ij}=\frac{\frac{1}{\left|P_{i}\right|}\underset{p_{i}\in P_{i}}{\sum }{\text{max}}_{p_{j}\in P_{j}}\left\{s\left(p_{i},p_{j}\right)\right\}+\frac{1}{\left|P_{j}\right|}\underset{p_{j}\in P_{j}}{\sum }{\text{max}}_{p_{i}\in P_{i}}\left\{s\left(p_{j},p_{i}\right)\right\}}{2}$$

where *P_i _*and *P_j _*are the sets of proteins *p_i _*and *p_j _*annotated to the probesets *i *and *j*, respectively.

Four topological measures were used to calculate the topological similarity scores *s*(*p_i_, p_j_*) between pairs of proteins *p_i _*and *p_j_*: 1) normalized geodesic distance; 2) Jaccard coefficient; 3) functional similarity; 4) a probabilistic common neighborhood similarity.

In order to describe the four topological similarity measures considered in this study, we first introduce some terms and notations. The network of the interactions is defined as graph *G = *(*V, E*) consisting of a set of nodes *V *and a set of edges *E *between them. *p_i _*and *p_j _*refer to proteins which are the nodes of the network; *N*(*p_i_*) and *N*(*p_j_*) are the neighbors of *p_i _*and *p_j _*respectively, and *N*(*p_i_, p_j_*) *= N*(*p_i_*)∩ *N*(*p_j_*).

##### Normalized geodesic distance

The normalized geodesic distance (NG) between two proteins *p_i _*and *p_j _*is defined as the normalized length of the shortest path, *l*(*path*(*p_i_, p_j_*)), from *p_i _*to *p_j _*, obtained by dividing *l*(*path*(*p_i_, p_j_*)) by the maximum of the shortest paths between all pairs of proteins. The similarity *s*(*p_i_, p_j_*) between two proteins is derived as 1 minus the normalized shortest path:

(8) $$s\left(p_{i},p_{j}\right)=1-\frac{l\left(path\left(p_{i},p_{j}\right)\right)}{\underset{p_{k},p_{r}\in V\left(G\right)}{\text{max}}\left\{l\left(path\left(p_{k},p_{r}\right)\right)\right\}}$$

##### Jaccard coefficient

The similarity measure *s*(*p_i_, p_j_*) based on the Jaccard coefficient (JA) [44] is defined as the ratio between the number of neighbors which two proteins share (common neighbors) and the total number of proteins they are connected to:

(9) $$s\left(p_{i},p_{j}\right)=\frac{\left|N\left(p_{i},p_{j}\right)\right|}{\left|N\left(p_{i}\right)\cup N\left(p_{j}\right)\right|}$$

##### Functional similarity

The functional similarity (FS) proposed in [23] measures the common neighborhood similarity of two proteins *p_i _*and *p_j _*in the network *G*, as:

(10) $$s\left(p_{i},p_{j}\right)=\frac{2\left|N\left(p_{i},p_{j}\right)\right|}{\left|N\left(p_{i}\right)-N\left(p_{j}\right)\right|+2\left|N\left(p_{i},p_{j}\right)\right|+\lambda_{ij}}\times \frac{2\left|N\left(p_{i},p_{j}\right)\right|}{\left|N\left(p_{j}\right)-N\left(p_{i}\right)\right|+2\left|N\left(p_{i},p_{j}\right)\right|+\lambda_{ji}}$$

where

(11) $$\lambda_{ij}=\text{max}\left(0,n_{avg}-\left(\left|N\left(p_{i}\right)-N\left(p_{j}\right)\right|+2\left|N\left(p_{i},p_{j}\right)\right|\right)\right)$$

and *n_avg _*is the average number of neighbors of proteins in the network. The term *λ_ij _*penalizes the score between protein pairs where at least one of the proteins has too few neighbors.

##### Probabilistic common neighborhood similarity

A probabilistic measure for the statistical significance (SC) of the common neighborhood configuration of two proteins *p_i _*and *p_j _*has been recently proposed [24]. The measure is defined as the negative logarithm of the probability of *p_i _*and *p_j _*having a certain number of common neighbors by random chance:

(12) $$s\left(p_{i},p_{j}\right)=-{\text{log}}_{10}\left(prob\left(N,\left|N\left(p_{i}\right)\right|,\left|N\left(p_{j}\right)\right|,\left|N\left(p_{i},p_{j}\right)\right|\right)\right)$$

*N *is the total number of proteins in the network, and *prob(N, |N(p_i_)|, |N(p_j_)|, |N(p_i_, p_j_)|) *is computed on the basis of the Hypergeometric distribution:

(13) $$prob\left(N,\left|N\left(p_{i}\right)\right|,\left|N\left(p_{j}\right)\right|,\left|N\left(p_{i},p_{j}\right)\right|\right)=\overset{\text{min}\left(\left|N\left(p_{i}\right)\right|,\left|N\left(p_{j}\right)\right|\right)}{\underset{k=\left|N\left(p_{i},p_{j}\right)\right|}{\sum }}\frac{\left(\begin{array}{c} \left|N\left(p_{i}\right)\right|\\ k \end{array}\right)\left(\begin{array}{c} \left|N\right|-\left|N\left(p_{i}\right)\right|\\ \left|N\left(p_{j}\right)\right|-k \end{array}\right)}{\left(\begin{array}{c} \left|N\right|\\ \left|N\left(p_{j}\right)\right| \end{array}\right)}$$

Thus, the higher the probability (13), the higher the value of *s(p_i_, p_j_) *is.

Equations (8), (9), (10) and (12) are finally used to derive *s_ij _*using Equation (7).

#### Correlation based similarity

Publicly available data from ten breast cancer microarray studies were extracted from GEO, selecting those with a medium to large sample size (Table 2). Redundant subjects were removed. All datasets were hybridized using Affymetrix U133 Genechips™ (HG-U133A and HGU133plus2) and were analyzed using A-MADMAN, an open source web application, which allows the retrieval, annotation, organization and meta-analysis of gene expression [45]. In particular, the software enables the integrative analysis of data obtained from different Affymetrix platforms through meta-normalization. Affymetrix chip definition files were used to annotate the arrays and gene expression intensity signal was normalized using RMA algorithm [36]. The resulting gene expression matrix collects the expression levels of 21921 probesets over 1586 biological samples.

**Table 2:** Breast cancer datasets used for the correlation based similarity

Datasets	Platform	Samples
GSE2034 [52]	HGU133A	286
GSE6532 [53]	HGU133A / HGU133plus2	225
GSE11121 [54]	HGU133A	200
GSE2990 [30]	HGU133A	189
GSE1456 [55]	HGU133A	159
GSE7390 [32]	HGU133A	155
GSE5460 [56]	HGU133plus2	127
GSE3494 [31]	HGU133A	110
GSE5847 [57]	HGU133A	95
GSE4922 [58]	HGU133A	40

Gene expression profiles over the ten datasets were compared using similarity measures based on Pearson correlation coefficient (PE), Spearman rank correlation coefficient (SP) and Mutual Information (MI), which provide a general measure to analyze dependencies in gene expression data [46-48].

Using both the Pearson and the Spearman correlation, the similarity *s_ij _*between two probesets *i *and *j *was defined as:

(14) $$s_{ij}=\left|\rho_{ij}\right|$$

To calculate Mutual Information we needed to quantize data on *L *intervals. There is no optimal solution to choose *L*, since it depends on data normalization and on the particular biological application [49]. As suggested in [47], heuristic lower/upper bounds on the number of intervals were considered [50,51]: *MI_low _*= ⌊1+log_2_*m*⌋ and *MI*_up _= √*m*, where *m *is the number of expression values. In our case, *L *= 25.

### Evaluation of the biomarker lists

Results were evaluated in terms of both stability of the biomarker lists obtained by the Canberra distance [7] and the accuracy performed by the perceptron classifier.

Given two ordered lists *T1 *and *T2 *of *p *ranked features, the Canberra distance between them is defined as:

(15) $$Ca\left(T1,T2\right)=\overset{p}{\underset{i=1}{\sum }}\frac{\left|\tau_{1}\left(i\right)-\tau_{2}\left(i\right)\right|}{\tau_{1}\left(i\right)+\tau_{2}\left(i\right)}$$

where *τ_1_(i) *and *τ_2_(i) *indicate the rank, i.e. the position, of the feature *i *in the ordered lists *T1 *and *T2*, respectively.

This measure is a weighted version of the Spearman's footrule which considers the variations in lower portions of the lists less relevant than those in the top [7]. A normalized version of this measure can be obtained by dividing the distance in (15) by its expected (average) value, approximated by *(log(4) -1)p + log(4) - 2 *for the complete lists. The normalized Canberra distance ranges between 0 (maximal stability) and 1.4 (maximal instability), with 1 in the case of randomly generated lists.

The average number of iterations needed by the perceptron in the algorithm is also considered as a good indicator of the ratio between the maximal norm of transformed vectors and the margin one can obtain in feature space. We consider this value as a measure of how much difficult is the transformed task.

Ranked feature lists obtained using different similarity matrices were evaluated both within datasets, i.e. comparing the 1000 different lists obtained using Bootstrap, and between datasets, i.e. comparing the global lists obtained by analyzing datasets GSE2990, GSE3494 and GSE7390. For the within dataset comparison, the Canberra distance was applied on the 1000 complete lists resulting from the Bootstrap resampling schema adopted by the classification algorithm. For the between datasets comparison, the Canberra distance was applied on the sublists of length *k*, with *k *corresponding to the minimum Canberra distance within dataset (average of the three values obtained for the three datasets). Finally, for the best performing similarity matrices, the union of the sublists of length *k *obtained using the three datasets, where *k *ranges from 1 to the maximum number of features (22207), was considered in order to quantify the possible lack of consistency of the global lists.

## Results

### Within dataset assessment

Table 3 reports the average normalized Canberra distance and classification accuracy for all the three breast cancer datasets and for all similarity matrices. Results are reported for the cases where prior information is not used (α = 0) and using for each similarity matrix the value of α (Equation 2) which minimizes the Canberra distance. For all the three datasets, all types of biological information are able to decrease the average normalized Canberra distance over the biomarker lists with respect to the standard classification approach. In particular, three types of prior knowledge are best performers in this task: Gene Ontology Biological Process (GO BP), Gene Ontology Molecular Function (GO MF) and protein-protein interactions codified by the normalized geodesic distance (PPI NG). For these three types of biological knowledge, the improvement in list stability, which ranges between 26% and 37%, is achieved without a corresponding loss in accuracy since this latter changes in a range between minus 2% to plus 3%.

**Table 3:** Classification performance within breast cancer datasets

	GSE2990	GSE3494	GSE7390
No prior	0.89 (95%) 7	0.93 (93%) 10	0.90 (98%) 6
GO BP	0.62 (93%) 15	0.63 (95%) 21	0.60 (96%) 13
GO MF	0.63 (93%) 17	0.68 (94%) 24	0.60 (97%) 15
PPI NG	0.57 (94%) 10	0.58 (96%) 14	0.53 (97%) 9
PPI JA	0.87 (95%) 7	0.91 (93%) 11	0.87 (97%) 7
PPI FS	0.88 (95%) 7	0.92 (95%) 11	0.88 (97%) 7
PPI SC	0.83 (95%) 8	0.86 (95%) 13	0.83 (96%) 8
PE	0.78 (95%) 49	0.89 (96%) 56	0.79 (96%) 37
SP	0.78 (95%) 48	0.89 (95%) 56	0.79 (95%) 38
MI	0.76 (91%) 130	0.80 (94%) 207	0.73 (94%) 131

Normalized Canberra distance between feature lists obtained for datasets GSE2990, GSE3494 and GSE7390, using the standard classification approach without prior knowledge integration and different prior knowledge based similarity matrices: Gene Ontology Biological Process (GO BP), Gene Ontology Molecular Function (GO MF), protein-protein interactions codified by the normalized geodesic distance (PPI NG), the Jaccard coefficient (PPI JA), the functional similarity (PPI FS), the probabilistic common neighborhood similarity (PPI SC), the Pearson correlation (PE), the Spearman rank correlation (SP) and the Mutual Information (MI). Predictive accuracy is indicated in brackets, whereas the number of iterations obtained by the classifier is reported below the other scores.

Table 3 also reports the number of iterations needed by the classification algorithm to reach convergence, averaged across the 1000 Bootstrap splits. Compared to other types of prior knowledge, the higher number of iterations are observed with the correlation (PE and SP) and Mutual Information (MI) based matrices, whereas PPI measures lead the classifier to reach convergence with a lower number of iterations, i.e. they improve class separability. However, except the normalized geodesic distance, all the other protein-protein interaction measures show the lowest gain in reproducibility.

### Between datasets assessment

Table 4 reports the average Canberra distance obtained by comparing datasets GSE2990 vs GSE3494, GSE2990 vs GSE7390, GSE3494 vs GSE7390, and the resulting average Canberra distance together with the average classification accuracy across the three datasets for *k *corresponding to the minimum Canberra distance within datasets (average of the three values obtained for the three datasets). GO BP, GO MF and PPI NG are confirmed as the best performing kinds of prior knowledge. In addition, MI based similarity matrix shows performance comparable to the former similarity matrices.

**Table 4:** Canberra distance and accuracy across breast cancer datasets

	*k*	GSE2990 vs GSE3494	GSE3494 vs GSE7390	GSE2990 vs GSE7390	Mean Canberra Distance	Mean Accuracy
No prior	4182	0.95	0.94	0.94	0.94	95%
GO BP	4268	0.63	0.65	0.65	0.65	95%
GO MF	3456	0.62	0.62	0.63	0.63	94%
PPI NG	8684	0.62	0.61	0.62	0.62	96%
PPI JA	22207	0.96	0.96	0.97	0.97	95%
PPI FS	22207	0.96	0.97	0.97	0.97	96%
PPI SC	22207	0.91	0.92	0.93	0.93	95%
PE	128	0.70	0.72	0.74	0.74	96%
SP	163	0.68	0.71	0.62	0.62	95%
MI	310	0.62	0.65	0.64	0.64	93%

Pair-wise Canberra distance between the three breast cancer datasets at different number of features selected according to the minimum Canberra distance within datasets, using the standard classification approach without prior knowledge integration and different prior knowledge based similarity matrices. The corresponding mean value and the mean accuracy obtained across the three datasets are also reported.

**Figure 1:**
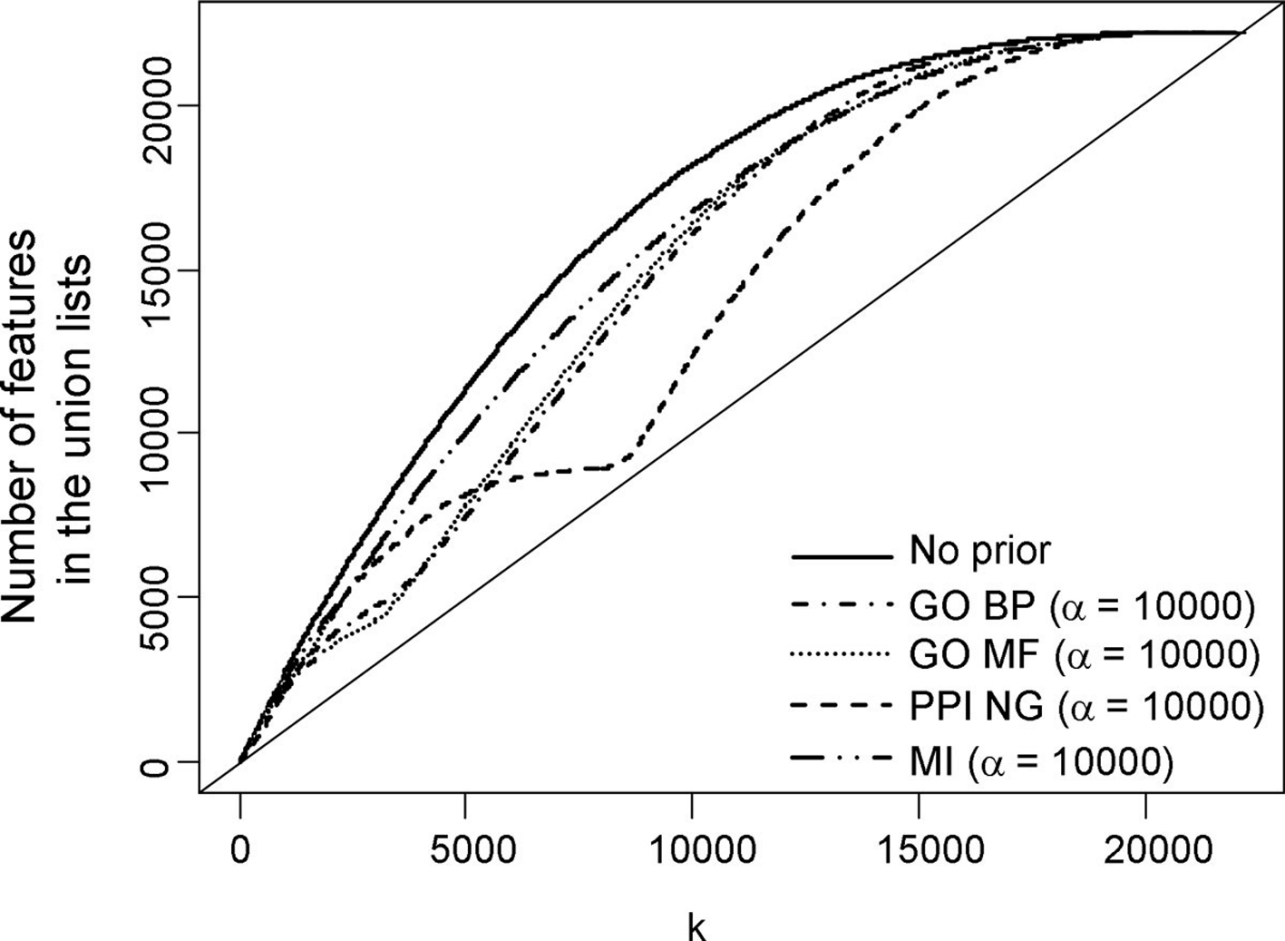
**Feature list stability**. Number of features in the union lists of length *k*, obtained by the standard classifier (No prior) and the integration of the best performing biological information: GO Biological Process (GO BP), GO Molecular Function (GO MF), protein-protein interactions codified by the normalized geodesic distance (PPI NG) and mutual information for gene expression data (MI).

In order to better assess the improvement highlighted in these four similarity matrices, we have looked at the size of the union sets of the biomarker lists of length *k *over all the three datasets (Figure 1). The more two lists are similar, i.e. containing the same features, the more the points of the curve are drawn near the diagonal. Compared with the standard approach, the union lists obtained from GO BP, GO MF and PPI NG are able to improve the feature ranking, but no meaningful improvements are evident for the similarity matrix obtained using MI similarity matrix. In particular, the two GO BP and GO MF based matrices provide the most stable union lists for *k *around 5000 features, whereas PPI NG matrix achieves the best performance for *k *around 9000 features.

## Discussion

The subject of the investigation in this paper is the effect of using information from the biological domain into a learning process with the aim of improving its general performance with respect to the stability of predicted biomarkers. State-of-the-art machine learning methods give solutions with empirically good performance in terms of accuracy. However, the stability of the selected biomarkers is also a very important issue. If an accurate system tends to select the same biomarkers in different independent experiments, then it is more likely that the selected biomarkers are the right ones.

In this work, we have integrated gene expression data and biological prior knowledge to enhance biomarker lists stability in a classification approach. In particular, we have compared the effect of incorporating different types of biological prior knowledge, like functional annotations, protein-protein interactions and expression correlation among genes in the learning process by evaluating biomarker list stability and classification accuracy.

Integrating prior knowledge is not an easy task since different types of information are represented in various data formats and stored in heterogeneous data structures. To do that, we have codified biological information into specific pair-wise similarity measures, chosen accordingly to the type of biological information used: semantic similarities for the annotations on GO, topology-based similarity measures for PPI and correlation for gene expression data. Feature space has then been mapped into a new space in which the more similar two features are, the more closely they are mapped. Our intuition is that when some features are strongly correlated to each other, for example because they belong to the same biological process, then they likely have similar importance and are equally relevant for the task at hand. In other words, the weight vector obtained for a classification task should have similar values on indices relative to similar genes. Following this intuition, we can bias the solutions to fulfill this property. Experimental results seem to support this intuition: our approach improves list stability, preserving high classification accuracy. In particular, three similarity matrices, based on GO BP, GO MF annotations and PPI NG, are the best performers in improving list stability. The lowest gain in biomarker list reproducibility is observed with the other matrices based on protein-protein interaction networks, whereas those based on correlation and mutual information achieve a better reproducibility but lead the classifier to reach convergence with a higher number of iterations.

In particular, the MI based matrix shows performance comparable to GO BP, GO MF and PPI NG based matrices when list stability is assessed between datasets.

This work compares the use of different biological information from genomic databases in the learning process. The technique proposed in this paper builds a kernel matrix from a similarity matrix, thus it can be used together with any kernel method (see [38] and references therein for a survey). In particular, it provides a standardized way to incorporate different types of biological knowledge in the kernel, with the only constraint that the information is codified by a similarity matrix.

Obtained results provide a starting point for additional experiments. As future work, we think it would be interesting to combine similarity matrices in order to obtain even more stable biomarkers, using for example the approach proposed by Bie et al. [28] to combine kernels. We believe that the power and potential of the proposed strategy will increase as the coverage and quality of biological databases improve.

## List of abbreviations used

Bp: Bayes point; AVE: average operation; ACC: prediction accuracy; DAG: direct acyclic graph; IC: Information Content; BMA: Best-Match Average approach; MICA: most informative common ancestor; NG: normalized geodesic distance; JA: Jaccard coefficient; FS: functional similarity; SC: probabilistic common neighborhood similarity; PE: Pearson correlation coefficient; SP: Spearman rank correlation; MI: Mutual Information.

## Competing interests

The authors declare that they have no competing interests.

## Authors' contributions

TS did the microarray and semantic/topological data analysis and drafted the manuscript. FA conceived the data integration algorithm, did the classification analysis and helped writing the manuscript. GDSM did the classification analysis. AB helped with the microarray data integration. BDC conceived and supervised the study and wrote the manuscript. All authors have read and approved the manuscript in its current form.

## References

[B1] Buness A Ruschhaupt M Kuner R Tresch A Classification across gene expression microarray studies BMC Bioinformatics 2009 10 453 10.1186/1471-2105-10-453 20042109 PMC2811711

[B2] Dudoit S Fridlyand J Speed TP Comparison of discrimination methods for the classification of tumors using gene expression data J Am Stat Assoc 2002 97 77 87 10.1198/016214502753479248

[B3] Simon R Development and validation of biomarker classifiers for treatment selection J Stat Plan Inference 2008 138 308 320 10.1016/j.jspi.2007.06.010 19190712 PMC2344143

[B4] Ein-Dor L Kela I Getz G Givol D Domany E Outcome signature genes in breast cancer: is there a unique set? Bioinformatics 2005 21 171 178 10.1093/bioinformatics/bth469 15308542

[B5] Solé X Bonifaci N López-Bigas N Berenguer A Hernández P Reina O Maxwell CA Aguilar H Urruticoechea A de Sanjosé S Comellas F Capellá G Moreno V Pujana MA Biological convergence of cancer signatures PLoS One 2009 4 e4544 10.1371/journal.pone.0004544 19229342 PMC2642727

[B6] Boulesteix AL Slawski M Stability and aggregation of ranked gene lists Brief Bioinform 2009 10 556 568 10.1093/bib/bbp034 19679825

[B7] Jurman G Merler S Barla A Paoli S Galea A Furlanello C Algebraic stability indicators for ranked lists in molecular profiling Bioinformatics 2008 24 258 264 10.1093/bioinformatics/btm550 18024475

[B8] Abeel T Helleputte T Van de Peer Y Dupont P Saeys Y Robust biomarker identification for cancer diagnosis with ensemble feature selection methods Bioinformatics 2010 26 392 398 10.1093/bioinformatics/btp630 19942583

[B9] Meinshausen N Buhlmann P Stability selection Journal of the Royal Statistical Society: Series B (Statistical Methodology) 72 417 473

[B10] Furlanello C Serafini M Merler S Jurman G Semisupervised learning for molecular profiling IEEE/ACM Trans Comput Biol Bioinform 2005 2 110 118 10.1109/TCBB.2005.28 17044176

[B11] Ambroise C McLachlan GJ Selection bias in gene extraction on the basis of microarray gene-expression data Proc Natl Acad Sci USA 2002 99 6562 6566 10.1073/pnas.102102699 11983868 PMC124442

[B12] Chuang HY Lee E Liu YT Lee D Ideker T Network-based classification of breast cancer metastasis Mol Syst Biol 2007 3 140 17940530 10.1038/msb4100180 PMC2063581

[B13] Rapaport F Zinovyev A Dutreix M Barillot E Vert JP Classification of microarray data using gene networks BMC Bioinformatics 2007 8 35 10.1186/1471-2105-8-35 17270037 PMC1797191

[B14] Li C Li H Network-constrained regularization and variable selection for analysis of genomic data Bioinformatics 2008 24 1175 1182 10.1093/bioinformatics/btn081 18310618

[B15] Yousef M Ketany M Manevitz L Showe LC Showe MK Classification and biomarker identification using gene network modules and support vector machines BMC Bioinformatics 2009 10 337 10.1186/1471-2105-10-337 19832995 PMC2774324

[B16] Tai F Pan W Incorporating prior knowledge of predictors into penalized classifiers with multiple penalty terms Bioinformatics 2007 23 1775 1782 10.1093/bioinformatics/btm234 17483507

[B17] Binder H Schumacher M Incorporating pathway information into boosting estimation of high-dimensional risk prediction models BMC Bioinformatics 2009 10 18 10.1186/1471-2105-10-18 19144132 PMC2647532

[B18] Kanehisa M Goto S Furumichi M Tanabe M Hirakawa M KEGG for representation and analysis of molecular networks involving diseases and drugs Nucleic Acids Res 2010 38 D355 D360 10.1093/nar/gkp896 19880382 PMC2808910

[B19] Ashburner M Ball CA Blake JA Botstein D Butler H Cherry JM Davis AP Dolinski K Dwight SS Eppig JT Harris MA Hill DP Issel-Tarver L Kasarskis A Lewis S Matese JC Richardson JE Ringwald M Rubin GM Sherlock G Gene ontology: tool for the unification of biology. The Gene Ontology Consortium Nat Genet 2000 25 25 29 10.1038/75556 10802651 PMC3037419

[B20] Chen X Wang L Integrating biological knowledge with gene expression profiles for survival prediction of cancer J Comput Biol 2009 16 265 278 10.1089/cmb.2008.12TT 19183004 PMC3198940

[B21] Haury AC Jacob L Vert JP Increasing stability and interpretability of gene expression signatures arXiv:1001.3109 2010 1 18

[B22] Pesquita C Faria D Bastos H Ferreira AE Falcão AO Couto FM Metrics for GO based protein semantic similarity: a systematic evaluation BMC Bioinformatics 2008 9 Suppl 5 S4 10.1186/1471-2105-9-S5-S4 18460186 PMC2367622

[B23] Chua HN Sung WK Wong L Exploiting indirect neighbours and topological weight to predict protein function from protein-protein interactions Bioinformatics 2006 22 1623 1630 10.1093/bioinformatics/btl145 16632496

[B24] Cho YR Zhang A Identification of functional hubs and modules by converting interactome networks into hierarchical ordering of proteins BMC Bioinformatics 2010 11 Suppl 3 S3 10.1186/1471-2105-11-S3-S3 20438650 PMC2863062

[B25] Keshava Prasad TS Goel R Kandasamy K Keerthikumar S Kumar S Mathivanan S Telikicherla D Raju R Shafreen B Venugopal A Balakrishnan L Marimuthu A Banerjee S Somanathan DS Sebastian A Rani S Ray S Harrys Kishore CJ Kanth S Ahmed M Kashyap MK Mohmood R Ramachandra YL Krishna V Rahiman BA Mohan S Ranganathan P Ramabadran S Chaerkady R Pandey A Human Protein Reference Database--2009 update Nucleic Acids Res 2009 37 D767 D772 10.1093/nar/gkn892 18988627 PMC2686490

[B26] Herbric R Graepel T Campbell C Bayes Point machines J Mach Learn Res 2001 1 245 279

[B27] Helleputte T Dupont P Buntine W, Grobelnik M, Mladenic D, Shawe-Taylor J Feature selection by transfer learning with linear regularized models Proceedings of the European Conference on Machine Learning and Knowledge Discovery in Databases: 7-11 September 2009 2009 Bled, Slovenia, Springer Berlin / Heidelberg 533 547

[B28] De Bie T Tranchevent LC van Oeffelen LM Moreau Y Kernel-based data fusion for gene prioritization Bioinformatics 2007 23 i125 i132 10.1093/bioinformatics/btm187 17646288

[B29] Barrett T Troup DB Wilhite SE Ledoux P Evangelista C Kim IF Tomashevsky M Marshall KA Phillippy KH Sherman PM Muertter RN Holko M Ayanbule O Yefanov A Soboleva A NCBI GEO: archive for functional genomics data sets-10 years on Nucleic Acids Res 2002 37 D885 D890 10.1093/nar/gkq1184 PMC3013736 21097893

[B30] Sotiriou C Wirapati P Loi S Harris A Fox S Smeds J Nordgren H Farmer P Praz V Haibe-Kains B Desmedt C Larsimont D Cardoso F Peterse H Nuyten D Buyse M Van de Vijver MJ Bergh J Piccart M Delorenzi M Gene expression profiling in breast cancer: understanding the molecular basis of histologic grade to improve prognosis J Natl Cancer Inst 2006 98 262 272 10.1093/jnci/djj052 16478745

[B31] Miller LD Smeds J George J Vega VB Vergara L Ploner A Pawitan Y Hall P Klaar S Liu ET Bergh J An expression signature for p53 status in human breast cancer predicts mutation status, transcriptional effects, and patient survival Proc Natl Acad Sci USA 2005 102 13550 13555 10.1073/pnas.0506230102 16141321 PMC1197273

[B32] Desmedt C Piette F Loi S Wang Y Lallemand F Haibe-Kains B Viale G Delorenzi M Zhang Y d'Assignies MS Bergh J Lidereau R Ellis P Harris AL Klijn JG Foekens JA Cardoso F Piccart MJ Buyse M Sotiriou C Strong time dependence of the 76-gene prognostic signature for node-negative breast cancer patients in the TRANSBIG multicenter independ-ent validation series Clin Cancer Res 2007 13 3207 3214 10.1158/1078-0432.CCR-06-2765 17545524

[B33] Weigel MT Dowsett M Current and emerging biomarkers in breast cancer: prognosis and prediction Endocr Relat Cancer 2010 17 R245 R262 10.1677/ERC-10-0136 20647302

[B34] Riccadonna S Jurman G Merler S Paoli S Quattrone A Furlanello C Supervised classification of combined copy number and gene expression data J Integr Bioinform 2007 4 74

[B35] Bioconductor Project http://www.bioconductor.org

[B36] Irizarry RA Hobbs B Collin F Beazer-Barclay YD Antonellis KJ Scherf U Speed TP Exploration, normalization, and summaries of high density oligonucleotide array probe level data Biostatistics 2003 4 249 264 10.1093/biostatistics/4.2.249 12925520

[B37] Freund Y Schapire RE Large margin classification using the perceptron algorithm J Mach Learn 1999 37 277 296 10.1023/A:1007662407062

[B38] Hofmann T Schoölkopf B Smola AJ Kernel methods in machine learning Ann Stat 2008 36 3 1171 1220 10.1214/009053607000000677

[B39] NetAffx™ Analysis Center http://ww.afymetrix.com/analysis/index.affx

[B40] Lord PW Stevens RD Brass A Goble CA Investigating semantic similarity measures across the Gene Ontology: the relationship between sequence and annotation Bioinformatics 2003 19 1275 1283 10.1093/bioinformatics/btg153 12835272

[B41] Couto FM Silva MJ Coutinho PM Measuring semantic similarity between gene ontology terms Data & Knowledge Engineering 2007 61 137 152 10.1016/j.datak.2006.05.00322403822

[B42] Lin D Jude W An information-theoretic definition of similarity Proc Int'l Conf Machine Learning: 24-27 July 1998; Madison, Wisconsin, USA 1998 Shavlik: Morgan Kaufmann 296 30422403011

[B43] De Las Rivas J Fontanillo C Protein-protein interactions essentials: key concepts to building and analyzing interactome networks PLoS Comput Biol 2010 6 e1000807 10.1371/journal.pcbi.1000807 20589078 PMC2891586

[B44] Jaccard P Étude comparative de la distribution florale dans une portion des Alpes et des Jura Bulletin de la Société Vaudoise des Sciences Naturelles 1901 37 547 579

[B45] Bisognin A Coppe A Ferrari F Risso D Romualdi C Bicciato S Bortoluzzi S A-MADMAN: annotation-based microarray data meta-analysis tool BMC Bioinformatics 2009 10 201 10.1186/1471-2105-10-201 19563634 PMC2711946

[B46] Steuer R Kurths J Daub CO Weise J Selbig J The mutual information: Detecting and evaluating dependencies between variables Bioinformatics 2002 18 231 240 10.1093/bioinformatics/18.suppl_2.S231 12386007

[B47] Priness I Maimon O Ben-Gal I Evaluation of gene-expression clustering via mutual information distance measure BMC Bioinformatics 2007 8 111 10.1186/1471-2105-8-111 17397530 PMC1858704

[B48] Guptaa N Aggarwal S MIB: using mutual information for biclustering gene expression data Pattern Recognition 2010 43 2692 2697 10.1016/j.patcog.2010.03.002

[B49] Daub CO Steuer R Selbig J Kloska S Estimating mutual information using B-spline functions - an improved similarity measure for analysing gene expression data BMC Bioinformatics 2004 5 118 10.1186/1471-2105-5-118 15339346 PMC516800

[B50] Sturges HA The choice of a class interval J Am Stat Assoc 1926 21 65 66

[B51] Law AM Kelton WD Simulation Modeling & Analysis 1991 New York: McGraw-Hill Co22400621

[B52] Wang Y Klijn JG Zhang Y Sieuwerts AM Look MP Yang F Talantov D Timmermans M Meijer-van Gelder ME Yu J Jatkoe T Berns EM Atkins D Foekens JA Gene-expression profiles to predict distant metastasis of lymph-node-negative primary breast cancer Lancet 2005 365 671 679 15721472 10.1016/S0140-6736(05)17947-1

[B53] Loi S Haibe-Kains B Desmedt C Lallemand F Tutt AM Gillet C Ellis P Harris A Bergh J Foekens JA Klijn JG Larsimont D Buyse M Bontempi G Delorenzi M Piccart MJ Sotiriou C Definition of clinically distinct molecular subtypes in estrogen receptor- positive breast carcinomas through genomic grade J Clin Oncol 2007 25 1239 1246 10.1200/JCO.2006.07.1522 17401012

[B54] Schmidt M Böhm D von Törne C Steiner E Puhl A Pilch H Lehr HA Hengstler JG Kölbl H Gehrmann M The humoral immune system has a key prognostic impact in node-negative breast cancer Cancer Res 2008 68 5405 5413 10.1158/0008-5472.CAN-07-5206 18593943

[B55] Pawitan Y Bjöhle J Amler L Borg AL Egyhazi S Hall P Han X Holmberg L Huang F Klaar S Liu ET Miller L Nordgren H Ploner A Sandelin K Shaw PM Smeds J Skoog L Wedrén S Bergh J Gene expression profiling spares early breast cancer patients from adjuvant therapy: derived and validated in two population-based cohorts Breast Cancer Res 2005 7 R953 R964 10.1186/bcr1325 16280042 PMC1410752

[B56] Lu X Lu X Wang ZC Iglehart JD Zhang X Richardson AL Predicting features of breast cancer with gene expression patterns Breast Cancer Res Treat 2008 108 191 201 10.1007/s10549-007-9596-6 18297396

[B57] Boersma BJ Reimers M Yi M Ludwig JA Luke BT Stephens RM Yfantis HG Lee DH Weinstein JN Ambs S A stromal gene signature associated with inflammatory breast cancer Int J Cancer 2008 122 1324 1332 17999412 10.1002/ijc.23237

[B58] Ivshina AV George J Senko O Mow B Putti TC Smeds J Lindahl T Pawitan Y Hall P Nordgren H Wong JE Liu ET Bergh J Kuznetsov VA Miller LD Genetic reclassification of histologic grade delineates new clinical subtypes of breast cancer Cancer Res 2006 66 10292 10301 10.1158/0008-5472.CAN-05-4414 17079448

